# Individual factors associated with L- and H-type Bovine Spongiform Encephalopathy in France

**DOI:** 10.1186/1746-6148-8-74

**Published:** 2012-05-30

**Authors:** Carole Sala, Eric Morignat, Nadia Oussaïd, Emilie Gay, David Abrial, Christian Ducrot, Didier Calavas

**Affiliations:** 1 ANSES-Lyon, 31 avenue Tony Garnier, Lyon cedex 7, 69364, France; 2 INRA-Theix, Lyon cedex 7, 69364, France

**Keywords:** Atypical bovine spongiform encephalopathy, L-BSE, H-BSE, Spatial analysis, Risk factors, France

## Abstract

**Background:**

Cattle with L-type (L-BSE) and H-type (H-BSE) atypical Bovine Spongiform encephalopathy (BSE) were identified in 2003 in Italy and France respectively before being identified in other countries worldwide. As of December 2011, around 60 atypical BSE cases have currently been reported in 13 countries, with over one third in France. While the epidemiology of classical BSE (C-BSE) has been widely described, atypical BSEs are still poorly documented, but appear to differ from C-BSE. We analysed the epidemiological characteristics of the 12 cases of L-BSE and 11 cases of H-BSE detected in France from January 2001 to late 2009 and looked for individual risk factors. As L-BSE cases did not appear to be homogeneously distributed throughout the country, two complementary methods were used: spatial analysis and regression modelling. L-BSE and H-BSE were studied separately as both the biochemical properties of their pathological prion protein and their features differ in animal models.

**Results:**

The median age at detection for L-BSE and H-BSE cases was 12.4 (range 8.4-18.7) and 12.5 (8.3-18.2) years respectively, with no significant difference between the two distributions. However, this median age differed significantly from that of classical BSE (7.0 (range 3.5-15.4) years). A significant geographical cluster was detected for L-BSE. Among animals over eight years of age, we showed that the risk of being detected as a L-BSE case increased with age at death. This was not the case for H-BSE.

**Conclusion:**

To the best of our knowledge this is the first study to describe the epidemiology of the two types of atypical BSE. The geographical cluster detected for L-BSE could be partly due to the age structure of the background-tested bovine population. Our regression analyses, which adjusted for the effect of age and birth cohort showed an age effect for L-BSE and the descriptive analysis showed a particular age structure in the area where the cluster was detected. No birth cohort effect was evident. The relatively small number of cases of atypical BSE and the few individual data available for the tested population limited our analysis to the investigation of age and cohort effect only. We conclude that it is essential to maintain BSE surveillance to further elucidate our findings.

## Background

Bovine spongiform encephalopathy (BSE) is a fatal neurodegenerative prion disease of cattle. The disease was first identified in the United Kingdom in 1986 before spreading to most European countries. Since epidemiological studies demonstrated the role of contaminated meat and bone meal (MBM), animal feed was considered the main source of BSE infection in cattle. The implementation of control measures that led in 2001 to a total ban on the use of MBM in animal feed allowed the BSE epidemic in Europe to be controlled [1]. However, the origin of the BSE agent remains uncertain: could it be due to a spontaneous sporadic bovine disease recycled into MBM or an adaptation of another transmissible spongiform encephalopathy (TSE) agent such as scrapie [2,3]? The results of studies on the transmission of BSE and scrapie to transgenic mice have not yet confirmed the ovine origin of BSE [4,5].

In 2003, two new BSE strains were identified: H-type (H-BSE) in France and L-type (L-BSE) in Italy [6,7]. These two strains differ both from each other and from classical BSE (C-BSE) by the atypical biochemical characteristics of the pathological prion protein, the ensuing brain lesions and the incubation period in animal models. Clinically, experimental challenges of cattle indicated dullness and amyotrophic changes that were not observed with C-BSE [8,9]. The first transmission trials in mice confirmed that H-BSE and L-BSE were distinct from both the BSE agent that caused the animal epidemic and from scrapie isolates [10,11].

However, recent experimental transmissions showed the capability of L-BSE to acquire C-BSE properties when transmitted to inbred mice or transgenic mice expressing the ovine prion protein (PrP) [12,13]. More recently, similar observations were made for H-BSE, which acquired C-BSE characteristics after serial passages in wild-type mice or the first passage in mice over-expressing the bovine PrP [14,15]. Moreover, the zoonotic potential of L-BSE was suspected as various isolates of L-BSE were successfully transmitted to non human primates [15,16] and to transgenic mice expressing human PrP [17,18]. H-BSE has not yet been successfully transmitted to mice transgenic for human PrP.

Such findings lead to the hypothesis that atypical BSEs are spontaneous sporadic diseases, a hypothesis compatible with very low prevalence (circa 1 case per million tested animals) and a worldwide distribution including those countries that are currently regarded as free of C-BSE [19]. However, while the C-BSE epidemic is under control, questions remain about atypical BSEs, their potential link with C-BSE and human prion diseases [13,19,20].

Until now, no epidemiological studies have been conducted to improve knowledge on atypical BSEs, probably due to the small number of cases. At this time, fewer than 60 atypical BSEs have been detected worldwide, and the prevalence of atypical BSE varies strongly within countries, half of the atypical cases being detected in France and Poland. Moreover, the geographical distribution of atypical cases did not appear homogenous in France, with an apparent over-representation of L-BSE cases in the mid-West.

The aim of this study was to investigate the epidemiological characteristics of the 23 atypical BSE cases detected in France from the beginning of the systematic screening for BSE, implemented in 2001, to late 2009. A spatial analysis was performed to identify clusters of atypical cases and a multivariate regression used to evaluate individual risk factors such as the age and birth cohort, previously identified as risk factors for C-BSE. For the reasons mentioned above, L-BSE and H-BSE were studied separately. Results are discussed in the light of more recent knowledge and compared to the epidemiology of C-BSE.

## Methods

### Cases and population data

In France, BSE monitoring was initiated in 1990. Initially based on the clinical surveillance of animals, it was progressively expended in January 2001 to include testing of all cattle over 30 months of age, whether dead-on-farm, euthanised or slaughtered for human consumption. Individual data on tested animals (date of birth, date of death, breed and farm location at the date of death) were extracted from the national database on BSE surveillance, a restricted access database maintained by the French agency for food, environmental and occupation health and safety in Lyon (Anses-Laboratory of Lyon).

The study included all bovines tested from 1^st^ January 2001 to 31^st^ December 2009 that were born and bred in France, for which the date of birth, date of death, breed and farm location were available. As all atypical cases were over eight years of age, the reference population was restricted to animals over eight years old at the time of testing. The homogeneity of the age distribution of the reference population was compared between *départements* using a non parametric test.

We included all the atypical cases, tested from 1^st^ January 2001 to 31^st^ December 2009 [21].

The characteristics of the L- and H-BSE cases identified from January 2001 to December 2009 were compared to those of C-BSE cases detected during the same period.

### Spatial analysis

Data on tested animals were aggregated per *commune*, the smallest administrative unit in France. In total there are 30,505 communes in mainland France. The median area is 10.8 km^2^ (range 0.04 to 759 km^2^). For each animal, the commune in which it was located at the time of death defined its position in geographic space.

We ran a separate spatial cluster analysis for H-BSE and L-BSE cases using the spatial scan statistic implemented within SaTScan v8.2.1.0. [22]. The spatial scan statistic tests whether the cases are evenly distributed among the background population, by comparing the risk of an animal being infected inside a moving window (changing in size and shape) to the risk outside this window. For each window, the likelihood is based on the ratio of the number of observed cases over the expected number of cases which is estimated inside and outside the window. The window that has the maximum likelihood ratio defines the most likely cluster. Then a large number of random datasets are generated (Monte-Carlo replications) under the null hypothesis of even distribution of cases, and the maximum likelihoods of the ratio of the number of observed cases over the expected number of cases inside and outside the window are calculated. If the maximum likelihood of the cluster of real data is ranked among the 5% highest, it is considered significant. We assumed that the number of L- and H-BSE cases followed a Poisson distribution and used an elliptical window, which is a more flexible approach than the more commonly-used circle [23]. To avoid detecting clusters that were too wide and therefore of limited value, and according to the recommendations for elliptic windows, we reduced the area of the ellipses to 20% of the tested population [23]. The number of Monte-Carlo replications was set to 999.

### Regression modelling

Logistic regression models were developed to quantify the association between age at death and birth cohort on the risk of being L- or H-BSE. The outcome variable was the status of the animals regarding atypical BSE (L-BSE, H-BSE, negative-tested animals) and the explanatory variables (age at death and birth cohort) were represented by restricted cubic spline functions with three knots located at the 10%, 50% and 90% of the percentile of age and cohort [24]. The Wald test was used to assess the association between each variable and the occurrence of atypical BSE types. Given the small number of cases, a bootstrap with 200 replications was run to assess the reliability of the effect of the significant variables.

The analyses were carried out in R software [25] and the contributed package RMS [26].

## Results

### Descriptive analyses

From 1^st^ January 2001 to 31^st^ December 2009, 12 L-BSE, 11 H-BSE and 699 C-BSE cases (173 over eight years of age) were detected in France. The reference population for the spatial analyses and regression models included the 6,138,710 animals over eight years of age tested negative for atypical BSE. All the atypical BSE cases were detected by active surveillance. Most of them (8/11 for H-BSE and 9/12 for L-BSE) were detected at rendering plants, in the same proportion as C-BSE cases (Fisher’s Exact Test, p = 0.12 and p = 0.09 respectively).

The age of H-BSE cases (median 12, range 8 to 18 years) and L-BSE cases (median 12, range 8 to 19 years) was significantly greater than the age of C-BSE cases (median 7, range 3 to 15 years), Wilcoxon rank test p <0.001. The age of H-BSE and L-BSE cases did not differ (Wilcoxon rank test p = 0.69). Table 1 shows the age distribution of BSE cases stratified by type.

**Table 1:** BSE cases detected in France, 1 January 2001 to 31 December 2009. Descriptive statistics of age (in years) at detection stratified by BSE type

BSE type	**minimum**	**1**^**st**^**quartile**	**median**	**mean**	**3**^**rd**^**quartile**	**maximum**
**C-BSE**	3.5	6.2	7.0	7.3	8.0	15.4
**L-BSE**	8.4	11.1	12.4	13.0	14.3	18.7
**H-BSE**	8.3	10.1	12.5	12.4	13.9	18.2

Unlike C-BSE cases, atypical cases were detected in beef cattle more often than in dairy cattle (81.8% for H-BSE and 83.3% for L-BSE *vs* 20.3% for C-BSE, exact binomial test: p-values < 0.001). None of the ten most tested beef cattle breeds had a greater risk of being atypical BSE considering animals over eight years of age (p-value = 0.43).

The prevalence of atypical BSE was low and there was no difference between H- and L-BSE, with 0.18 and 0.20 cases respectively per 100,000 tested animals when considering only animals tested over eight years old (p-value = 1). Comparatively, the prevalence of C-BSE was of 2.81 cases per 100,000 tested animals over eight years old for the study period.

**Figure 1:**
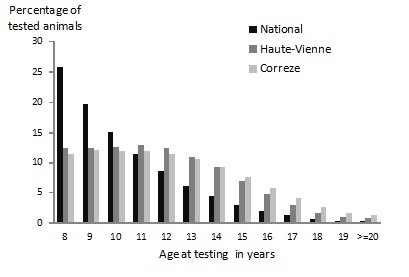
Proportion of bovines over eight years old tested per age at national level comparatively to the Haute-Vienne and Correze ‘départements’.

The age structure of the tested population differed significantly from that of the national population in two adjacent *départements*: Haute-Vienne and Corrèze, which had a similar age structure (Figure 1).

### Spatial analysis

**Figure 2:**
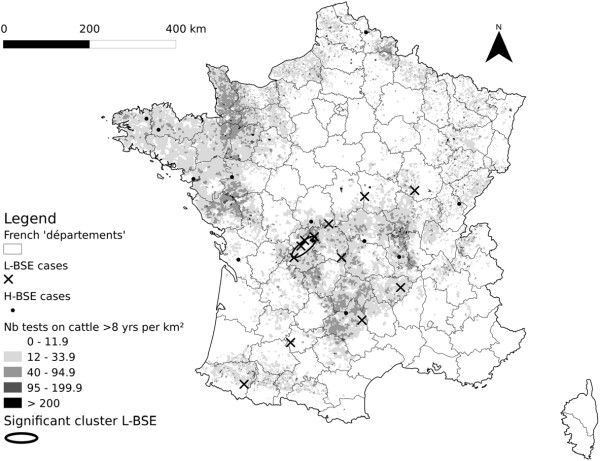
Geographical location of L-BSE and H-BSE cases and significant L-BSE cluster.

The spatial scan statistic highlighted one significant elliptic cluster for L-BSE (p-value = 0.03) located in mid-West France (Figure 2). The cluster was an ellipse of approximately 36 kilometres length and 12 kilometres width that included four cases and 29,359 negative bovines. No significant cluster was detected for H-BSE.

### Regression modelling

For L-BSE, our logistic regression model showed a significant association between the occurrence of the disease and the age (p-value = 0.001) but no association with the birth cohort (p-value = 0.15). The bootstrap run confirmed the association between L-BSE and age (p-value = 0.04).

For H-BSE, the model did not show any significant association with age or cohort (p-value = 0.73 and p-value = 0.58, respectively).

**Figure 3:**
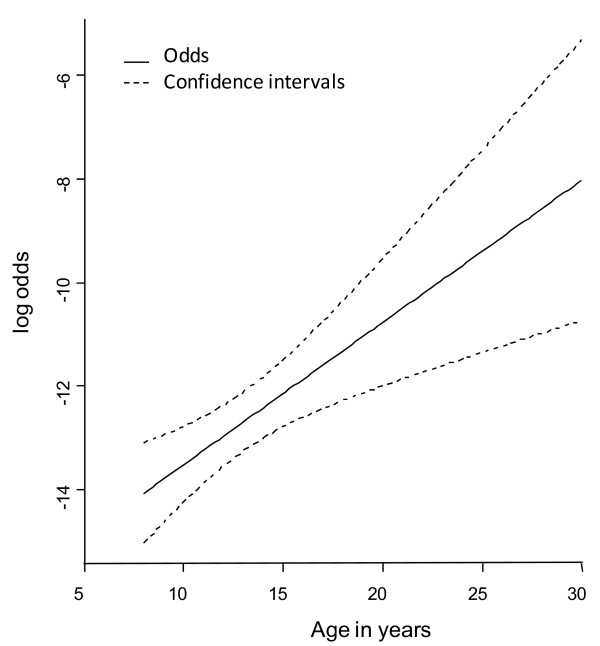
Relationship between age and L-BSE risk.

As the relationship between the occurrence of L-BSE and age was linear (Wald test: χ_2_ = 0, df = 1, p-value = 0.98), an estimate of the effect could be given by a simplified model with a linear relationship of age as the only covariate. The estimate of the slope was 0.28, which corresponds to an increase in the risk of L-BSE detection of 32% for every one year increment in age (Figure 3).

## Discussion

We identified an elliptical cluster of L-BSE cases in the mid-west of France and an association between age and the risk of being identified as an L-BSE case. Our spatial analyses and regression analyses found no such associations for H-BSE.

The statistical power of the study was limited by the very small number of cases detected (11 H-BSE and 12 L-BSE cases) and the scarcity of information available, which is restricted to the individual data routinely registered in BSE surveillance databases. No specific investigation was conducted to look for other risk factors, such as feed, as was the case for C-BSE [27,28].

The results of our spatial analyses and regression modelling were consistent with the descriptive analyses which showed only age as an individual risk factor for L-BSE. Despite the fact that the age distribution of the H-BSE and L-BSE cases did not differ significantly, no age effect was evident for H-BSE. This may have been due to the relatively small number of cases available for analysis. Nevertheless, the age effect for L-BSE and the distribution of the age of atypical cases differed from those of C-BSE where the risk of detection has been shown to increase up to 5–6 years of age, after which time it decreases [29-31].

All the atypical cases detected in France were over eight years of age, as were all the cases in the countries having identified atypical BSE cases. Only one Japanese L-BSE case was detected in a 23-month-old bovine, but this diagnosis could not be formally confirmed [32,33].

Compared to dairy cattle, beef cattle were over-represented in the atypical case group, whatever the type. This is consistent with the fact that the average age at detection for atypical cases was around 12 years and that, considering animals over eight years of age, the average age at testing of beef cattle is significantly greater than that of dairy cattle (11.2 years *vs* 9.5 years, p-value <0.001). Moreover, beef cattle represented 80% of those animals that were greater than 12 years of age at the time of testing. The over-representation of beef cattle cases is probably due to a difference in the age structure in these two subpopulations.

Concerning the results of the spatial analysis, it was not possible to take into account the age structure of the tested population by aggregating the data per age group, given the relatively small number of L- and H-BSE cases.

We ran the spatial analysis using the location of the animals at death while previous spatial analyses of C-BSE cases were performed with the birth location of the animals [34]. In fact, we had no prior hypothesis about the source, ways and age at infection (under the hypothesis of transmissible disease). Moreover the *commune* of birth of 3.7% of the tested animals was not available. Additionally only two H-BSE cases and four L-BSE cases died in another *commune* than their location at birth; among them one H-BSE and two L-BSE cases moved to contiguous *communes*, the others moved to another *département.* The results of the spatial analysis showed a significant cluster for L-BSE located in the mid-west of France. A previous spatio-temporal analysis of C-BSE showed that this area was at higher risk of C-BSE only for the animals born from July 1994 to June 1995 [35], and that was not the period of birth of the L-BSE cases included in the significant cluster in our analysis.

The significant cluster identified for L-BSE could be due to a particular age structure of the local subpopulation of animals tested when they were over eight years of age. The age structure of cattle in Haute-Vienne, where the cluster was located, differed significantly from that of the national population, as well as the one of Corrèze, in which no atypical cases were detected despite the structure of the tested population being similar in these two *départements* (Figure 1). Nevertheless, the number of L-BSE cases identified in Haute-Vienne (three cases per 133,689 tested) and in Corrèze (0 cases per 127,652 tested) were within the confidence interval of the overall national prevalence of L-BSE observed in France applied to the population tested in these two areas, as the standardised prevalence per 100,000 tested animals over eight years old was 0.3 (95% CI 0.0 to 3.5) in Haute-Vienne and 0.4 (95% CI 0.0 to 3.7) in Corrèze.

The prevalence of H- and L-BSE was low compared to C-BSE in the same period and within the same tested population. However, the prevalence of atypical BSEs varies widely worldwide, probably varying with the number of aged cattle tested and the age structure of this sub-population. This may explain the high number of L-BSE cases detected in Poland, where the proportion of animals over eight years of age is around 40% of the tested population^1^, and the prevalence of L-BSE for the 2002–2007 period has been estimated to be 7.7 cases per 100,000 tested animals. Moreover, compared to C-BSE, the prevalence of atypical BSE may be underestimated as the rapid tests used in routine testing for BSE were evaluated for classical BSE, for which the brain location of the pathological PrP is not the same in animals with atypical forms of BSE [7,13,21].

## Conclusion

In conclusion, our results are compatible with the hypothesis that atypical BSEs, or at least L-BSE, may represent a natural ageing process in cattle similar to that observed in some human diseases. However, the results of transmission studies investigating the potential role of atypical BSE in the C-BSE epidemic and its possible transmission to humans induces caution. We conclude that it is essential to maintain BSE surveillance to further elucidate our findings.

## Endnote

^a^The proportion of animals tested when over eight years old was evaluated from the BSE surveillance data of the 2002–2006 period published by M.Polak.

## Author’s contribution

CS participated in the design of the study, performed the descriptive analysis and drafted the manuscript. EM performed the modelling analysis and participated in the drafting of the manuscript. NO performed the spatial analysis. EG participated in the design of the study and helped in performing the spatial analysis. CD and DC coordinated the study, participated in the design of the study and helped to draft the manuscript. All authors read and approved the final manuscript. This work was funded by Anses.
